# Study on the Properties of Solidified Silt Based on Microbially Stimulated Recycled Hardened Cement Powder

**DOI:** 10.3390/ma18112575

**Published:** 2025-05-30

**Authors:** Xihui Yin, Chuanjiang Tian, Jintao Hong, Qiwei Zhan, Xinyu Wang, Wanying Dong

**Affiliations:** 1 No. Nine Engineering Co., Ltd. of CCCC First Highway Engineering Co., Ltd., Guangzhou 511300, China; 2 School of Civil Engineering and Architecture, Jiangsu University of Science and Technology, Zhenjiang 212100, China; 3 School of Material Science and Engineering, Southeast University, Nanjing 211189, China

**Keywords:** recycled hardened cement powder (RHCP), carbon sequestration, spore-forming bacillus, solidified sludge

## Abstract

The carbon emissions from the cement industry account for approximately 8% of global carbon emissions, which exerts significant pressure on the environment. In this paper, the microbial-induced calcium carbonate precipitation (MICP) technology was introduced into the carbonization modification research of recycled hardened cement powder (RHCP), and the carbon sequestration performance of RHCP under different pressures was studied. The physicochemical properties of the carbonated products were characterized by microscopic testing methods, and the carbon sequestration mechanism under different pressures was obtained. Subsequently, carbonated RHCP (C-RHCP) was tested as a partial cement substitute for solidified sludge to evaluate its mechanical and durability properties. The results show that when the pressures were 0.3 and 0.5 MPa, the carbon sequestration capacity of RHCP was relatively good, reaching 59.14 and 59.82 g/kg, respectively. Since the carbon sequestration amounts under the two pressures were similar, and considering the energy consumption, in this study, a reaction pressure of 0.3 MPa was selected to prepare C-RHCP. Compared with pure cement, the 28-day unconfined compressive strength (UCS) of the sludge cured with 30% C-RHCP increased by 12.08%. The water stability coefficient of the solidified sludge in the C-RHCP group was greater than 1 after soaking for 7, 14, and 21 days, while the water stability coefficient of the cement group decreased to 0.92 at 14 days. After 20 freeze–thaw cycles, the mass losses of the cement group, the RHCP group, and the C-RHCP group were 31.43%, 38.99%, and 33.09%, respectively. This research not only provides an environmentally friendly strategy for the resource utilization of RHCP but also pioneers a new synergistic model that combines microbial mineralization with the modification of industrial solid waste. It demonstrated significant scientific value and engineering application prospects in reducing carbon emissions in the cement industry and promoted sustainable geotechnical engineering practices based on the “waste–waste” principle.

## 1. Introduction

As a reliable construction material, the demand for concrete increased continuously with the development of urbanization [1,2,3]. Due to geological disasters and human construction activities, the amount of abandoned concrete has been increasing year by year. In China, the annual construction waste generated amounts to nearly 3 billion tons, with abandoned concrete accounting for about 30% [4]. Recycled hardened cement powder (RHCP) has been produced through the crushing, screening, and grinding of discarded concrete [5]. Its particle size is generally less than 75 μm, making it a byproduct of recycled aggregate production [6]. According to related studies, approximately 5.4 million tons of recycled powder are produced annually in China [7]. Most of these powders were buried underground, which not only wasted resources but also imposed a serious burden on the environment [8]^.^ Due to the low reactivity of recycled powder, it is difficult to utilize on a large scale. Research had shown that when recycled powder was incorporated into concrete, its workability decreased by 12.2–36.73% [9,10], and its mechanical performance decreased by 32.17–62.84% [11]. In recent years, many scholars found that reducing the particle size of RHCP and using CO_2_ for carbonization treatment effectively addressed this issue. Zhao et al. [12] found that when the particle size of RHCP was reduced, its specific surface area and pozzolanic activity were further enhanced. This could accelerate the early hydration of cement paste and shorten its setting time. Tang et al. [13] found that when the particle size was less than 75 μm, the pore structure was significantly improved. In recent years, studies have reported that RHCP separated from discarded concrete also had good CO_2_ absorption capacity [14]. After carbonization treatment, both aggregates and powders could be better utilized in concrete production [15,16,17]. During the carbonization reaction, CO_2_ reacted with calcium-containing minerals in cementitious materials such as calcium hydroxide (CH), ettringite (AFt), etc., resulting in the formation of calcium carbonate (CaCO_3_) and amorphous silica gel (SiO_2_·nH_2_O) [18,19]. H. Mehdizadeh et al. [20] found that the addition of carbonated cement paste powder (CCPP) could increase the compressive strength of cement paste mortar (CCPPM) by about 20% and reduce water permeability by about 50%. Liu et al. [21] argued that the carbonization process affected the proportion of gel sites in calcium silicate hydrate (C-S-H). The characteristics of the silicon chains in C-S-H changed with increasing carbonization degree until the calcium silicate hydrate was completely transformed into silica gel, which helped enhance the pozzolanic properties of RHCP.

Sludge has been defined as the fine-grained portion of clayey soil or soil with a plasticity index less than 4 [22]. Due to the non-ideal particle size distribution of clay, the interparticle bonding strength is weak. When sludge is relatively dry, it is brittle and not easily compacted. However, when its moisture content is high, its resistance to permeability is poor. These performance limitations make it challenging for it to be widely used in engineering applications [23,24]. Therefore, before engineering applications, it is necessary to treat it with solidification, which could effectively improve various properties of sludge [25]. Commonly used solidifying agents in engineering include cement, lime, and geopolymer binders [26,27,28]. The solidification effect they produce is primarily due to physical or chemical reactions between the internal binder materials and substances within the soil [29]. The pozzolanic effect of volcanic ash in cement is the primary reason for its solidifying action. Additionally, cement hydration could increase the pH of the soil, accelerating the dissolution of its internal mineral components. C-S-H and C-A-S-H gels with cementitious properties are generated in this process, which can improve soil performance [30]. Following the introduction of the national dual-carbon strategy, researchers are no longer limited to traditional curing materials such as cement. Instead, industrial by-products combined with CO_2_ are used to develop new curing agents as alternatives to cement, contributing to the dual-carbon strategy [31,32]. Wang et al. investigated a coupling technique for CO_2_ carbonation with industrial by-products, using ternary MgO-CaO-FA and binary MgO-FA mixtures to stabilize soil [33,34]. The results showed that CO_2_ carbonation combined with active MgO-CaO-FA mixtures was highly effective and reliable in enhancing the strength properties of soil samples. Additionally, the binary MgO-FA mixture significantly improved the environmental resistance of the stabilized sludge, demonstrating better durability. In addition, their team proposed two methods to assess the CO_2_ absorption of soil stabilized with active magnesium–lime–fly ash [35]. The results indicated that the thermogravimetric method was more accurate than the weight gain method. Furthermore, the carbonation effect increased with longer carbonation times and higher binder contents. These findings provided a microscopic perspective on the feasibility of using industrial by-products combined with CO_2_ for stabilizing sludge. In addition to traditional curing agents and new industrial by-product-CO_2_ curing agents, organic modifiers such as plant fibers and starch were incorporated into Yellow River silty soil to improve soil cohesion. Polymeric short fibers were added to the stabilized sludge to enhance its strength [36,37].

The carbon sequestration properties of RHCP under different pressures were studied by means of microbiome-induced calcium carbonate precipitation. X-ray diffraction (XRD) and Fourier transform infrared spectroscopy (FTIR) were employed to investigate the mineral composition of carbonated RHCP (C-RHCP). Scanning electron microscopy coupled with energy-dispersive spectroscopy (SEM-EDS) was used to observe the microstructure and elemental distribution of the sample. Combined with thermogravimetric (TG) analysis, the carbon sequestration capacity of C-RHCP was quantitatively evaluated. Simultaneously, the effect of different substitution levels of C-RHCP and RHCP, replacing 10–40% of cement, on the unconfined compressive strength (UCS) of solidified sludge was investigated. The optimal substitution level was determined to explore the impact of different moisture contents and curing ages on the UCS of dredged sludge. Subsequently, water stability, wet–dry cycling, and freeze–thaw cycling experiments were conducted to determine the long-term performance of solidified sludge. This study innovatively explored the carbonization performance of industrial solid waste RHCP by combining it with a microbial-induced calcium carbonate precipitation method under different pressures. The presence of microbes significantly enhanced the carbonization effect and increased the pozzolanic activity of the RHCP, supporting its use as a partial replacement for cement in curing agents. Additionally, the method featured simple process conditions and a green production approach, offering a new avenue for the utilization of RHCP.

The microbial-induced calcium carbonate precipitation (MICP) technology was innovatively introduced into the carbonization modification research of RHCP, and the enhancement mechanism of microbial synergy on the carbonization performance of RHCP under varying pressure conditions was systematically revealed for the first time. A microbial-pressure coupled activation system was constructed, through which the carbonation efficiency and pozzolanic activity of RHCP were significantly improved, thereby overcoming the limitations of low efficiency and high energy consumption inherent in conventional carbonization technologies. This study not only was recognized as providing an environmentally friendly strategy for the resource utilization of RHCP but also was established as pioneering a novel synergistic model integrating microbial mineralization with industrial solid waste modification. Significant scientific value and engineering application prospects were demonstrated in reducing carbon emissions in the cement industry and advancing sustainable geotechnical engineering practices based on the “waste treatment with waste” principle.

## 2. Materials and Methods

### 2.1. Materials

The raw materials selected for this experiment mainly included recycled hardened cement powder (RHCP), cement, sludge, and natural microbial strains. RHCP was obtained by grinding and sieving test waste concrete specimens. The cement was produced by Jiangsu Helin Cement Co., Ltd. (Zhenjiang, China), and its label was P·O 42.5. The sludge came from the G312 National Highway reconstruction and expansion project, and it was used after precipitation, drying, and crushing. In this experiment, the readily available natural microbial strain Bacillus M.K. was used, which had excellent capability to induce calcium carbonate precipitation. It was cultured in LB medium in our laboratory.

**Figure 1:**
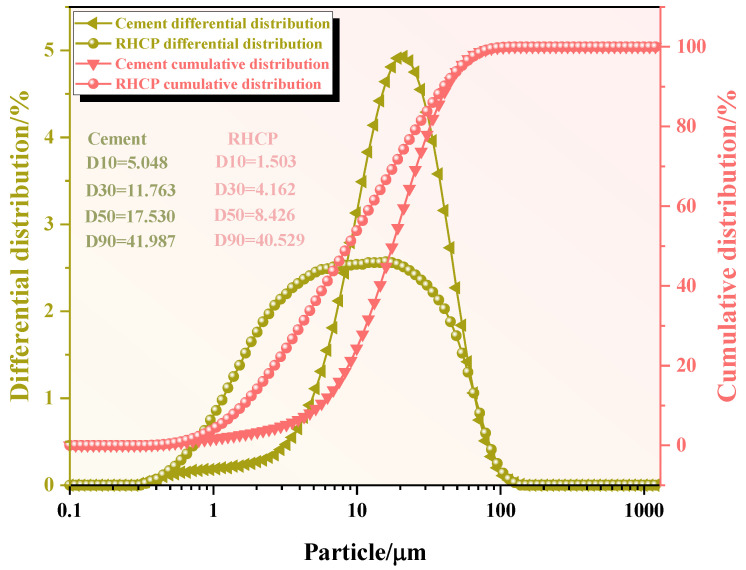
Particle size distribution of cement and RHCP.

**Figure 2:**
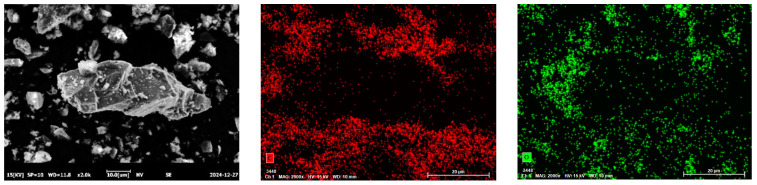

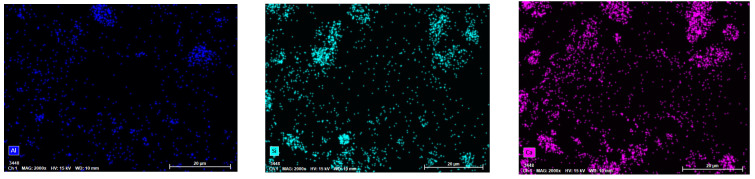
EDS spectra of RHCP.

The chemical compositions of the RHCP and cement were determined using X-ray fluorescence spectrometry (XRF), and the results are shown in Table 1. From Table 1, it could be seen that RHCP and cement mainly consisted of SiO_2_, CaO, Fe_2_O_3_, and Al_2_O_3_. The CaO content in RHCP reached a maximum of 63.46%, while the highest SiO_2_ content in cement was 61.501%. The particle size distributions of RHCP and cement were tested using a laser particle size analyzer. The test results are shown in Figure 1. From Figure 1, it was observed that both RHCP and cement had relatively small particle sizes and a uniform distribution, mainly ranging between 1 and 200 μm. The elemental composition of RHCP was determined using EDS, and the test results are shown in Figure 2. As shown in Figure 2, RHCP was mainly composed of Ca, C, O, Al, and Si, which was consistent with the results obtained from the XRF analysis.

### 2.2. Experimental Procedure

**Figure 3:**
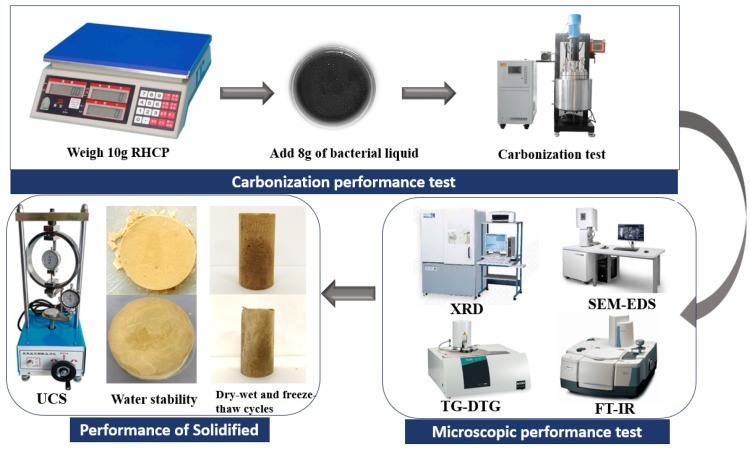
Test flowchart.

First, 10 g of RHCP was weighed and then mixed thoroughly with 8 g of bacterial solution that had been shaken and cultured for 24 h. The samples were then placed in a reaction vessel, and 99.9% pure carbon dioxide was introduced to expel excess air. After expelling the air from the reaction vessel, the apparatus was sealed, and pure CO_2_ was continuously introduced. Once the pressure reached 0.1, 0.3, 0.5, 0.7, and 0.9 MPa, the gas flow was stopped, and the sample was allowed to react for 12 h. After the reaction, the sample was removed, dried, ground, and set aside for future use. Next, 200 g of sludge and 24 g of cement were weighed and mixed with 100 g of water, stirring until uniform. The slurry was poured into molds coated with Vaseline and compacted uniformly, and then the specimens were placed in a standard curing box for curing. After a certain curing period, the specimens were taken out to measure their mechanical properties and long-term performance. The specific process was shown in Figure 3.

### 2.3. Test Methods

#### 2.3.1. Mineral Phase Composition

In this experiment, the crystal phases in C-RHCP were analyzed using an X-ray diffractometer (model TD-3500, Dandong Tongda Science & Technology Co., Ltd., Dandong, China). Samples were analyzed using Cu Kα target radiation, with a scanning range from 5° to 85°, and a scanning rate of 0.3° per second step. In contrast to the metallic and non-metallic crystalline phases detected by XRD, functional groups could be analyzed in substances for supplementary analysis by Fourier transform infrared spectroscopy (FTIR-650S, Cialan, Zhengzhou, China). The measurements were conducted using the potassium bromide (KBr) pellet method, with a total of 64 scans including background and sample collection. The equipment resolution and mirror speed were set at 2.0 cm^−1^ and 0.625, respectively.

#### 2.3.2. Microscopic Morphology and Elemental Composition

The microscopic morphology of C-RHCP was observed using a COXEM-30 scanning electron microscope (SEM) (COXEM, Daejeon, Republic of Korea). Due to the poor conductivity of the sample, it was necessary to first apply a uniform coating of gold powder to its surface. Subsequently, the sample was placed in the SEM, and the interior was evacuated to a vacuum state. The microscope lens was then opened to begin observation. Simultaneously, the QUANTAX X flash energy dispersive spectrometer (EDS) (Bruker, Billerica, MA, USA) could be activated to determine the elemental composition.

#### 2.3.3. Quantitative Characterization of Carbon Sequestration

Due to the high content of CaO in RHCP, it had a certain potential for carbon sequestration. CaO, MgO, and K_2_O could be directly reacted with CO_2_ to form carbonate products, achieving CO_2_ sequestration. Although SO_3_ was not directly reacted with CO_2_, it could be reacted with calcium compounds present in the material to form calcium sulfate (CaSO_4_), which would reduce the carbonation potential of RHCP. Based on the content of CaO, SO_3_, MgO, and K_2_O, the maximum theoretical CO_2_ sequestration capacity of RHCP (Qt) was estimated to be approximately 226.3 g/kg [38,39].

(1) $$Qt=\left[0.785\left(CaO-0.7SO_{3}\right)+1.01MgO+0.93K_{2}O\right]\times 100\%$$

The carbon sequestration capacity of C-RHCP was measured using a thermogravimetric analyzer (model HTG-2) produced by Beijing Hengjiu Co., Ltd. (Beijing, China). The sample, weighing approximately 10 mg, was placed in an alumina crucible and then heated from room temperature to around 1000 °C at a rate of 10 °C/min. The weight loss of the sample at different temperatures was recorded using a precision electronic balance. The carbon sequestration amount of C-RHCP was quantitatively characterized according to Formula (2) [40,41]. W_1_ refers to the temperature at which calcium carbonate first begins to decompose in the DTG curve and the mass remaining on the TG. W_2_ refers to the TG mass remaining at the end of decomposition of calcium carbonate in the DTG curve.

(2) $${CO}_{2uptake}=\frac{W_{1}-W_{2}}{W_{2}}\times 100\%$$

#### 2.3.4. Mechanical and Long-Term Performance Testing

##### Unconfined Compressive Strength (UCS)

In this experiment, the UCS of stabilized sludge (SS) was measured using a fully automated unconfined compression strength testing instrument. UCS of SS was calculated according to Formula (3) [29]. F_M_ represented the maximum pressure value of the instrument. S represented the contact area between the sample and the instrument.

(3) $$P=\frac{F_{M}}{S}$$

##### Water Stability

The SS was formed into disc-shaped specimens with a diameter of 62 mm and a height of 20 mm, followed by standard curing. After being cured for a specified age T, it was immersed in pure water. As water gradually infiltrated, it eroded the cracks and pores on the surface of SS, weakening the interaction forces between particles. Therefore, the water stability of SS was evaluated using the water stability coefficient K, calculated as shown in Formula (4) [42]. S_w_ was the UCS of the specimen soaked for 1 day after being cured for T-1 days. S was the UCS of the specimen after curing for the specified age T.

(4) $$K=S_{w}/S$$

##### Dry–Wet Cycles

Seven specimens were prepared with dimensions of Φ = 39.1 mm, h = 80 mm (1 control group, 3 experimental groups, and 3 control groups). After the specimen was formed, the mold was removed for use. The UCS of the control group was measured, and the masses of the experimental and control groups were recorded. Then, the experimental groups were placed in an oven and dried at 60 °C for 24 h. Meanwhile, the control groups were subjected to standard curing, immersed in water for 23 h, and then dried and weighed. After completing the cycles 1, 3, 5, 7, and 9, UCS and mass were measured to determine the mass and strength loss rates under different cycling conditions [43]. The calculation method was shown in Formulas (5) and (6). S_n_ represented the average relative mass loss rate after n cycles. M_s_ is the average dry mass of the test sample. W_i,s_ is the average dry mass loss of the sample during the ith cycle. M_c_ is the average dry mass of the control sample. W_i,c_ is the average dry mass loss of the control sample during the ith cycle. S_i_ is the strength loss rate after the ith cycle. f_28_ is the compressive strength of the specimen after 28 days of curing. f_n_ is the average compressive strength of three specimens after n cycles.

(5) $$S_{n}=\sum_{i=1-n}\left(\frac{W_{i,s}}{M_{s}}-\frac{W_{i,c}}{M_{c}}\right)$$

(6) $$S_{i}=\frac{f_{28}-f_{n}}{f_{28}}\times 100\%$$

##### Freeze–Thaw Cycling

First, the samples were weighed, and their UCS was measured after soaking for 24 h. Subsequently, they were placed into the freeze–thaw cycling test machine for freeze–thaw cycling experiments. The calculation methods of mass loss rate M_n_ and compressive strength loss rate T_n_ after n cycles are shown in Formulas (7) and (8). m_28_ is the mass of the sample after curing for 28 days. m_n_ is the mass of the sample after n cycles. S_28_ is the compressive strength of the sample after curing 28 days. S_n_ is the compressive strength of the sample after n cycles

(7) $$M_{n}=\frac{m_{28}-m_{n}}{m_{28}}\times 100\%$$

(8) $$T_{n}=\frac{S_{28}-S_{n}}{S_{28}}\times 100\%$$

## 3. Results and Discussion

### 3.1. The Mineral Phase Composition of C-RHCP

**Figure 4:**
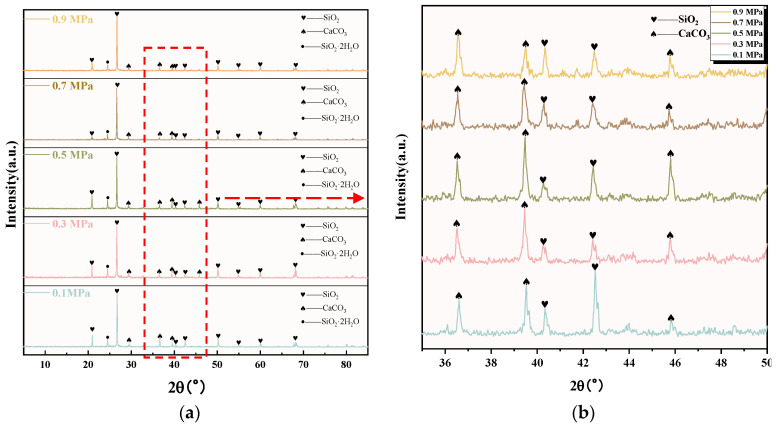
XRD pattern of RHCP with different carbonization pressures. (**a**) 5–85°; (**b**) 35–50° enlarged view.

The XRD images of C-RHCP under different pressures are shown in Figure 4. It can be seen from Figure 4a that C-RHCP was mainly composed of silicon dioxide (SiO_2_), calcium carbonate (CaCO_3_), and amorphous silica gel (SiO_2_·2H_2_O). The diffraction peaks of SiO_2_ mainly appeared at 20.91°, 26.75°, 40.62°, 40.74°, 50.18°, 54.91°, 59.94°, and 68.04°. Compared with the diffraction peak of CaCO_3_ and SiO_2_·2H_2_O, the diffraction peak intensity of SiO_2_ reached 5000, which was much higher than the diffraction peak of the other two substances. Because RHCP contained a higher proportion of silica materials with low reactivity, this made it less likely to participate in reactions. Therefore, SiO_2_ was its main component. To better contrast the crystalline phase changes of C-RHCP under different pressures, the images between 35 and 50° were zoomed in for detailed observation. The diffraction peaks of CaCO_3_ mainly appeared at 36.6°, 39.5°, and 45.86°. It can be seen from Figure 4b that the height of the CaCO_3_ diffraction peak at 39.5 ° and 45.86° became stronger with the increase in pressure. When the pressure exceeded 0.5 MPa, the crystallinity decreased. After exceeding 0.5 MPa of pressure, its crystallinity actually decreased. It was indicated that pressure to a certain extent enhanced the carbon sequestration effectiveness of RHCP [44]. However, the activity of microorganisms was also affected when the pressure increased. Excessive pressure could actually diminish their carbon sequestration efficiency [45]. The diffraction peak of amorphous silica gel was observed around 24°. Unlike other siliceous materials, it was endowed with a certain volcanic ash activity that could provide an active source of silica [46], which was advantageous for the subsequent development of concrete strength.

**Figure 5:**
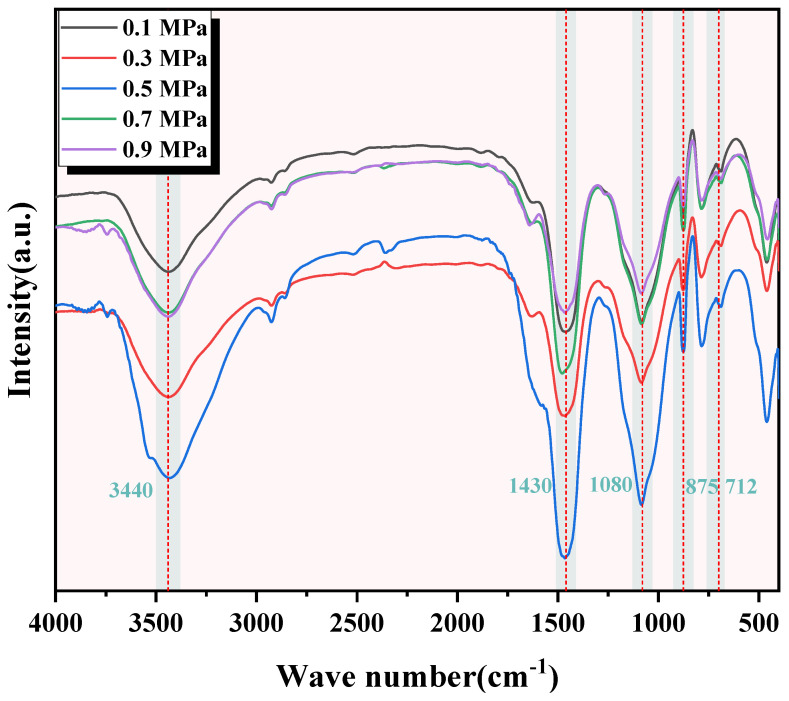
FTIR of RHCP under different carbonization pressures.

The FTIR test results of C-RHCP under different pressures are shown in Figure 5. It can be seen from Figure 5 that the images of C-RHCP under different carbonization pressures were basically the same. This indicated that the types of products formed after its reaction were essentially the same. At the wavenumber of 3440 cm^−1^, there was a significantly broad vibration peak observed in all five, corresponding to the symmetric and antisymmetric stretching vibrations of hydroxyl groups (-OH) in adsorbed water within the products [47]. Poorly crystalline phases or bound water was indicated to be present within C-RHCP based on diffusion characteristics. The vibration peak around 1080 cm^−1^ corresponded to the stretching vibration of Si-O [48]. This was because the Si-O vibration peak originally around 970 cm^−1^ for C-S-H shifted to a higher wavenumber, indicating the formation of amorphous silica gel. It was consistent with the results analyzed in XRD, indicating that the formation of amorphous silica gel would promote the development of strength in SS. There were three distinct vibration peaks at wave numbers 1430, 875, and 712 cm^−1^, which corresponded to the formation of calcium carbonate in the product. The vibration modes of the three were, respectively, the C-O asymmetric stretching vibration (v^3^) band, the out-of-plane bending vibration of C-O (v^2^), and the in-plane bending vibration of C-O (v^4^) [49,50]. Calcium carbonate formation was effectively detected by XRD and FTIR but could not be quantitatively characterized by them. In this paper, TG was used to quantitatively characterize the content of calcium carbonate in C-RHCP to determine the carbonization effect under different pressures.

### 3.2. Quantitative Analysis of Carbon Fixation

**Figure 6:**
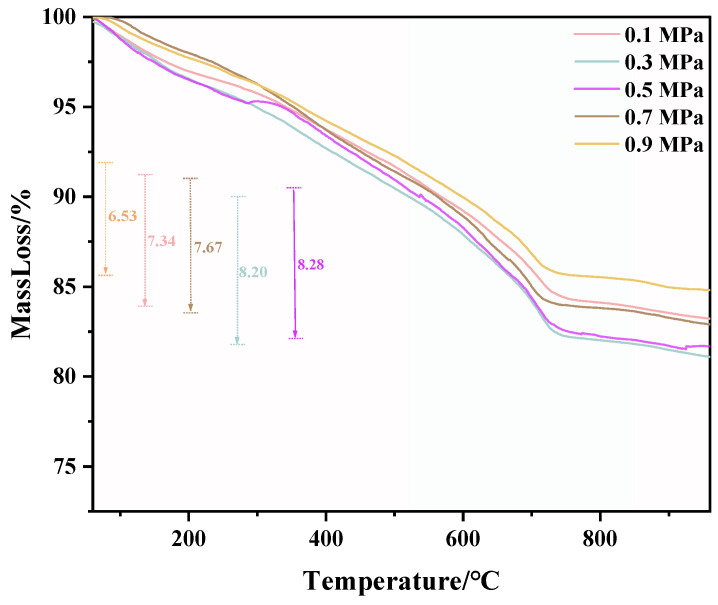
TG of RHCP with different carbonization pressures.

**Figure 7:**
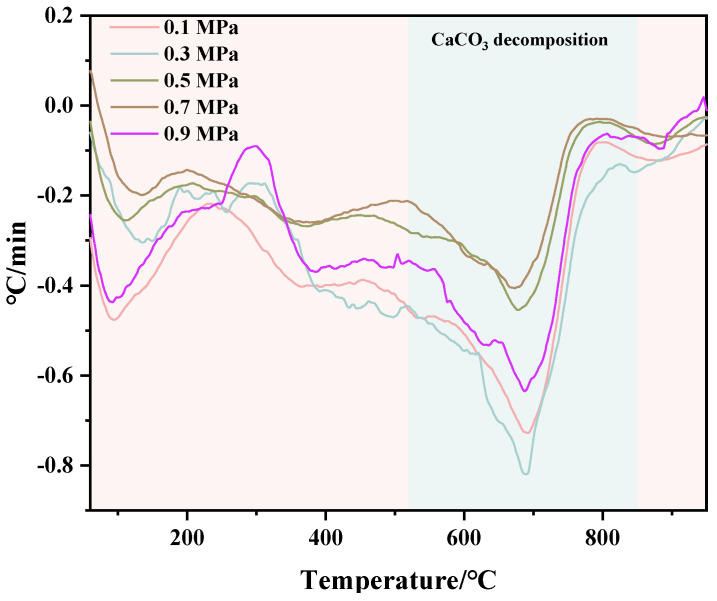
DTG of RHCP with different carbonization pressures.

TG and DTG test results of C-RHCP under different pressures are shown in Figure 6 and Figure 7, respectively. From Figure 6, it can be seen that there was a significant weight loss step between 500 and 900 °C, which corresponded to the decomposition of CaCO_3_. However, the specific decomposition temperature range could not be determined precisely. The exact decomposition temperature range needed to be confirmed through the DTG image. In Figure 7, it can be observed that there was an endothermic peak at different decomposition temperatures of the sample, with a noticeable peak at 520–840 °C. It represented that the decomposition temperature of CaCO_3_ produced in this experiment was between 520 and 840 °C [51]. Due to the fixed amount of CO_2_ already sequestered in RHCP, this portion needed to be deducted. According to Formula (2), the carbon fixation in RHCP under different pressures was calculated, and the specific results are shown in Table 2. Compared to RHCP, the carbon fixation of C-RHCP increased by 112.89%, indicating significant effectiveness of microbial-induced carbonization. Research indicated that carbonic anhydrase (CA) in microbes could enhance the solubility of carbon dioxide in water [52]. Under normal conditions, the hydration conversion factor of CO_2_ was only 1.3 × 10^−1^ s^−1^, whereas CA could increase this reaction rate to 1 × 10^6^ s^−1^ [53]. When the pressure increased from 0.1 MPa to 0.3 MPa, the carbon sequestration rate increased by 27.47%. The increase in pressure also contributed to the enhanced dissolution of CO_2_. However, there was no significant change in carbon sequestration when the pressure increased from 0.3 MPa to 0.5 MPa. Due to the increased pressure, although CO_2_ dissolution was enhanced, the activity of microorganisms was reduced, leading to RHCP’s carbonization capacity showing insignificant changes [54]. As the pressure continued to increase, the carbon fixation capacity was actually weakened to a certain extent. It indicated that the impact of pressure on microbial activity outweighs its effect on CO_2_ dissolution. Higher energy consumption was also induced by increased pressure. Hence, 0.3 MPa was considered the most appropriate reaction pressure.

### 3.3. The Microscopic Morphology of C-RHCP

**Figure 8:**
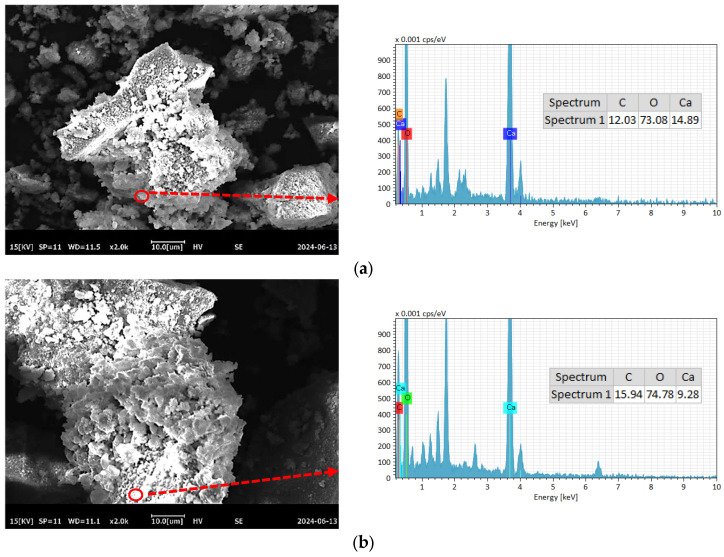

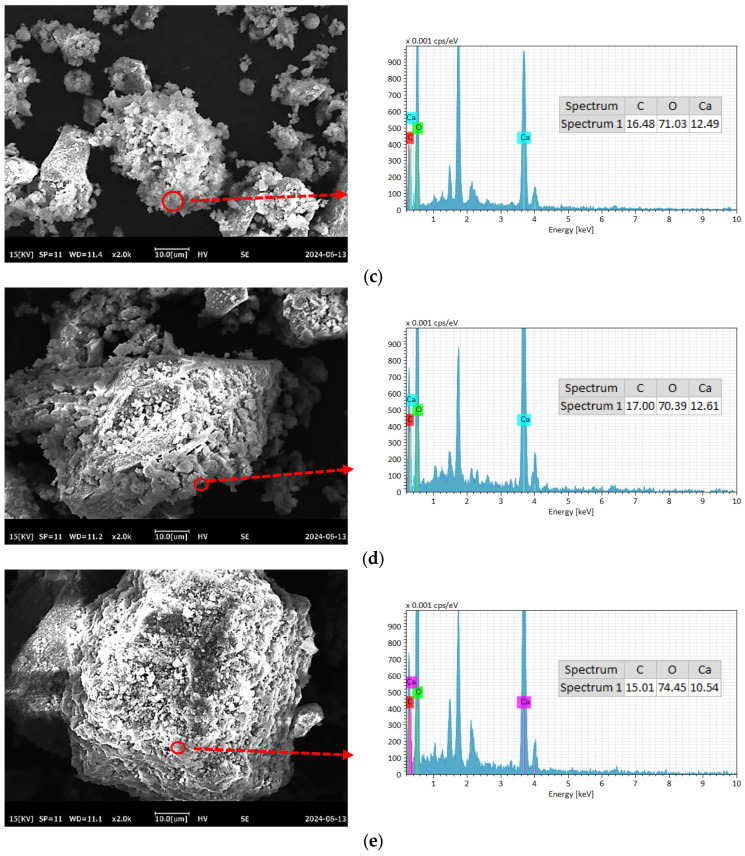
SEM-EDS of RHCP with different carbonization pressures. (**a**) 0.1 MPa, (**b**) 0.3 MPa, (**c**) 0.5 MPa, (**d**) 0.7 MPa, (**e**) 0.9 MPa.

To further compare the differences in C-RHCP under different pressures, SEM-EDS was employed for testing, and the test results are shown in Figure 8. As can be seen from Figure 8a–e, a lot of reactant precipitation was generated on the surface of C-RHCP. The elements of one point were determined by EDS, and the results showed that it was mainly composed of Ca, C, and O. This was due to the effect of calcium carbonate precipitation induced by microorganisms, which formed nucleation points on the surface of RHCP and promoted the precipitation of calcium carbonate [55]. At the same time, it also provided nuclear sites for the formation of hydration products, which could accelerate the hydration reaction [56]. When the pressure was 0.3 and 0.5 MPa, it was evident in Figure 8b,c that more sediment was produced on the surface of RHCP, covering almost the entire particle surface. This showed that the carbonization effect was better at these two pressures, which was consistent with the results found in TG. When the pressure increased to 0.9 MPa, the particle surface precipitation decreased significantly, as shown in Figure 8e. Although the solubility of CO_2_ would be increased with the raise of pressure, it was difficult to ensure the activity of microorganisms under high pressure [54]. At the same time, its role in inducing calcium carbonate precipitation would be significantly weakened. Therefore, fewer surface deposits were observed on C-RHCP under higher pressure conditions, as shown in Figure 8e. According to research reports, calcium carbonate deposited by microbial methods was characterized by small crystal particles and a large specific surface area [57]. This allowed it to effectively fill some of the internal pores of RHCP, thereby enhancing its mechanical properties.

### 3.4. The Interactions Between Bacillus, RHCP, and CO_2_

**Figure 9:**
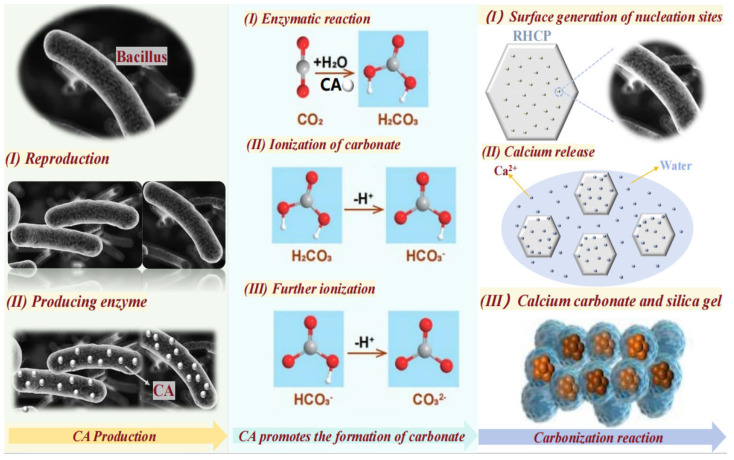
Action mechanism diagram of Bacillus, RHCP, and CO_2_.

The interactions between Bacillus, RHCP, and CO_2_ were divided into three main steps, as shown in Figure 9. First, Bacillus was diffused in water and rapidly produced carbonic anhydrase (CA). CA was then gradually dispersed into the liquid, significantly accelerating the reaction between CO_2_ and water, which promoted the formation of carbonic acid. The carbonic acid was gradually dissociated in water, losing hydrogen ions and ultimately forming the carbonate ions needed for the reaction. Finally, RHCP released some calcium ions in water, which reacted with the carbonate ions to produce fine calcium carbonate. Due to the action of Bacillus, numerous nucleation sites were formed on the surface of RHCP, facilitating the formation of calcium carbonate. This process filled the pores within and on the surface of RHCP, resulting in superior mechanical properties when used as a curing agent compared to uncarbonated RHCP. Additionally, the nucleation sites provided by Bacillus favored the formation of hydration products and amorphous silica gel, which positively impacted the mechanical and durability properties of the stabilized sludge.

### 3.5. Mechanical Performance Analysis of SS

#### 3.5.1. Dosage

**Figure 10:**
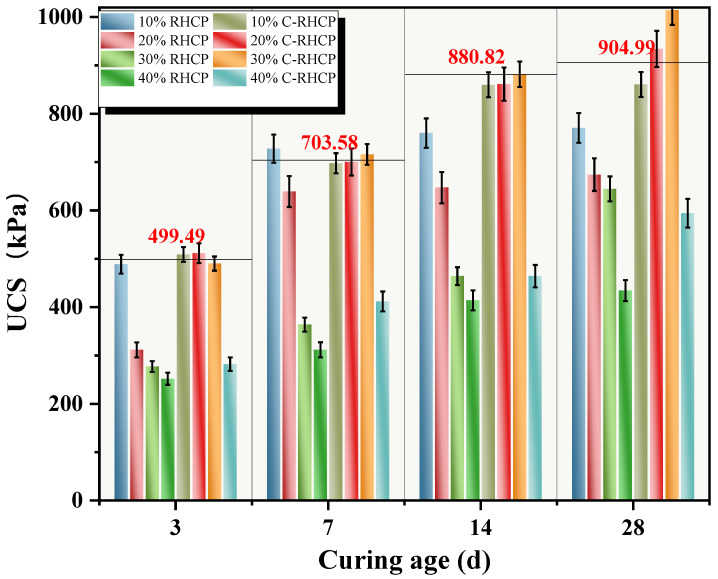
UCS of CS with different substitution rates.

RHCP and C-RHCP were used to replace part of the cement, aiming to achieve the goals of resource recycling and low carbon. Regenerated micro-powder is derived from construction waste. Its micro-aggregate filling effect and pozzolanic activity (reacting with cement hydration products to form C-S-H gel) can enhance the density and later strength of concrete, while reducing the amount of cement used to lower carbon emissions. Carbonization treatment generates calcium carbonate and silica gel through the reaction of CO_2_ with Ca(OH)_2_ in micro-powder, enhancing the material’s activity and microscopic compactness, sequestering CO_2_ and optimizing the early hydration rate and long-term durability. Application shows that when replacing 10–40% of cement, the strength and impermeability of concrete were close to or better than the benchmark, and the reduction of CO_2_ per cubic meter can reach 80–100 kg. It has dual advantages in terms of environment and performance, and it helps the green transformation of the building materials industry The sludge itself had high moisture content, low density, and large porosity, so its physical properties were not effectively improved by a low dosage of cement [58]. When the dosage of cement was increased, there was a positive correlation with the UCS of the sludge. However, due to the different on-site construction environments and methods of cement mixing, it was challenging for the two to exhibit a linear relationship. In this experiment, RHCP and C-RHCP were used to partially replace cement as curing agent, and UCS was used as evaluation index. UCSs of different ages under different substitution amounts are shown in Figure 10. It can be seen from Figure 10 that the UCS of SS under different replacement amounts increased with the extension of curing age. When the age of SS was 3 days, 7 days, 14 days, and 28 days, the UCS of cement SS with different substitution amounts were 499.49, 703.58, 880.82, and 904.99 kPa, respectively. From Figure 10, it can be seen that the UCS of C-RHCP-solidified sludge with substitution rates of 10–30% showed no significant decrease at various curing ages. However, at a substitution rate of 40%, its UCS was significantly reduced, much lower than that of the cement control group. On the contrary, at curing ages of 3 days and 7 days, the UCS of RHCP solidified sludge with only a 10% substitution rate showed no significant decrease. Significant decreases were observed at other curing ages and substitution rates. This indicated that the unmineralized RHCP replacement of cement was less effective than the C-RHCP. The solidification of sludge with C-RHCP replacing cement is feasible.

#### 3.5.2. Curing Age

**Figure 11:**
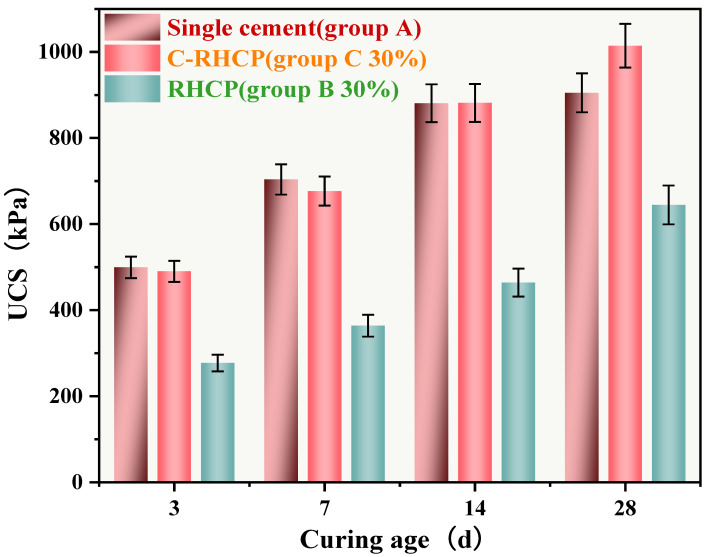
UCS of CS at different maintenance ages.

Due to the favorable mechanical performance of C-RHCP-solidified sludge at a 30% substitution rate, this substitution level was used in all subsequent experiments. The UCS of single-cement (Group A), 30% cement replacement with RHCP (Group B), and C-RHCP (Group C) sludges are shown in Figure 11. From Figure 11, it can be observed that with the increase in curing period, the UCS of Groups A, B, and C all exhibited an increasing trend. The UCSs for Group A at different ages (3, 7, 14, 28 days) were 499.49, 703.58, 880.82, and 904.99 kPa, respectively. For Group B, the UCSs were 277.14, 363.77, 464.04, and 644.50 kPa, respectively. Compared to Group A, the UCSs of Group B decreased by 44.52%, 48.30%, 47.32%, and 28.78% respectively. This indicated that as the curing period increased, the influence of RHCP on strength began to diminish. However, there remained a significant difference in overall strength between the two, indicating that RHCP did not contribute positively to strength development. The UCSs for Group C at different ages were 490.09, 676.73, 881.61, and 1014.32 kPa, respectively. Compared to Group A, the UCS of Group B could achieve 98.12%, 96.18%, 100.08%, and 112.08% of the cement control group’s values. This indicated that C-RHCP had a certain promotional effect on the UCS of SS. When carbonated RHCP replaced cement, the strength of the SS did not significantly decrease and slightly increased at a curing period of 28 days.

In the early stage of hydration (3–7 days), the rapid hydration of C_3_S and others in the cement generated C-S-H gel and Ca(OH)_2_. Meanwhile, the active SiO_2_/Al_2_O_3_ of RHCP initiated the pozzolans reaction, and the dual cementation formed a dense matrix. In the mid-stage of rehydration (7–14 days), the pre-carbonated CaCO_3_ in C-RHCP filled the capillary pores, and CO_2_ reacted with the residual Ca(OH)_2_ to form nano-CaCO_3_, further optimizing the pore structure. However, the intensity stagnated after 14 days. This might have been because the soluble silicon-aluminum phase and free Ca(OH)_2_ were basically exhausted, and the kinetics of the pozzolana-carbonization reaction was controlled by Ca^2+^ diffusion.

#### 3.5.3. Moisture Content

**Figure 12:**
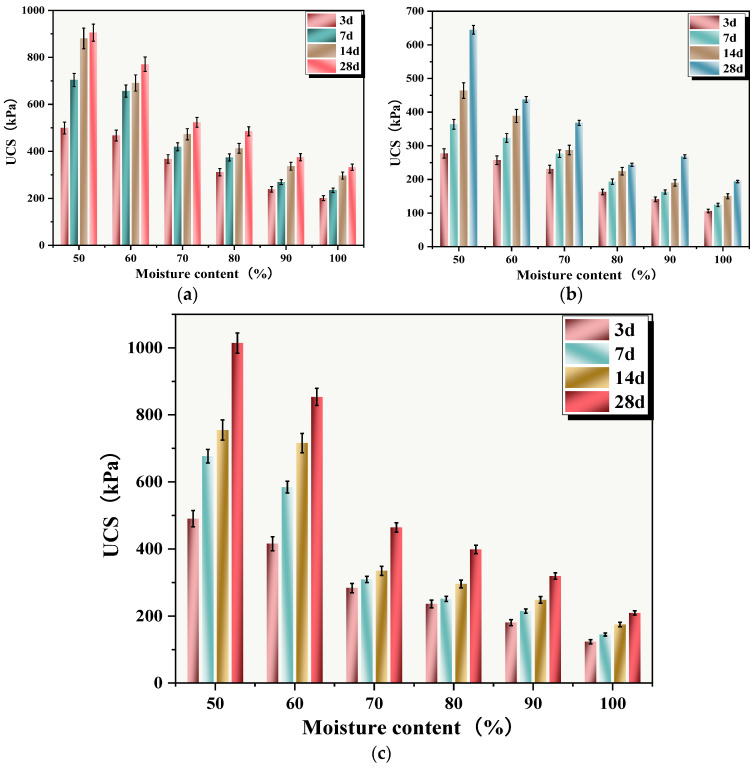
UCS of cement-cured silt with different moisture contents. (**a**) Single cement; (**b**) 30%RHCP; (**c**) 30%C-RHCP.

The relationship between sludge moisture content and UCS of SS is shown in Figure 12. When the moisture contents were 50%, 60%, 70%, 80%, 90%, and 100%, the UCSs of SS cured for 28 days were 904.99, 770.72, 523.60, 485.32, 374.97, and 332.64 kPa, respectively. It could be seen that sludge moisture content had a significant impact on the UCS of SS, with increasing moisture content being highly detrimental to the UCS growth of SS. The UCS of C-RHCP solidified sludge with 30% cement replacement is shown in Figure 12c. At a curing period of 28 days, the UCSs of the SS were 1014.32, 853.71, 464.05, 398.79, 319.56, and 209.46 kPa, respectively. When the moisture content did not exceed 60%, C-RHCP replacement of cement had a promoting effect on the UCS of SS, increasing it by 12.8% and 10.76%, respectively. Conversely, it hindered the growth of UCS. The UCS of RHCP-solidified sludge with 30% cement replacement is shown in Figure 12b. After 28 days of curing, the UCS values were 644.50, 437.73, 368.15, 243.63, 267.92, and 163.65 kPa, respectively. There was a noticeable decrease in UCS, with reductions of 28.78%, 43.20%, 29.68%, 49.8%, 28.5%, and 37.03%, respectively. The solidification effect of 30% C-RHCP-solidified sludge was similar to that of the single-cement control group and significantly better than the 30% RHCP group. This indicated that the UCS was inversely proportional to moisture content, influenced by multiple factors [59]. When the moisture content significantly exceeded the water required for the reaction, the excess water occupied space that could be considered as pores and defects within the solidification system [60]. This led to a decrease in UCS. The more excess water present, the lower the UCS. Excess water dispersed within the particles and reduced their binding strength, thereby decreasing the overall strength. Within the solidified sludge system, there were soluble hydration products such as calcium hydroxide and hydrated calcium aluminate. Despite their low solubility, these also had a certain negative impact on solidification strength.

When RHCP and C-RHCP are used as curing agents, the increase in the unconfined compressive strength of the specimens mainly stems from the optimization of the microstructure. Active SiO_2_ and Al_2_O_3_ are coarsely present in RHCP. They react with the hydration product Ca(OH)_2_ of cement to form additional C-S-H gels, enhancing the compactness of the cementation network. Due to the carbonization reaction of C-RHCP, nanoscale CaCO_3_ crystals are formed on its surface, which rigidly fill the pores and reduce defects. Meanwhile, carbonization consumes free Ca(OH)_2_ and reduces the risk of erosion of the structure by alkaline pore liquid. In addition, nano-scale calcium carbonate can also provide nuclear sites for the hydration of cement, promoting the hydration of cement. This will accelerate the growth of the cementitious phase, and the carbonization products form an interlocking structure with the unreacted particles, strengthening the interface transition zone. The synergistic effect of the two significantly enhances the density of the matrix and the bonding force between particles, which is macroscopically manifested as an increase in compressive strength.

### 3.6. Long-Term Performance Analysis of SS

#### 3.6.1. Water Stability

**Figure 13:**
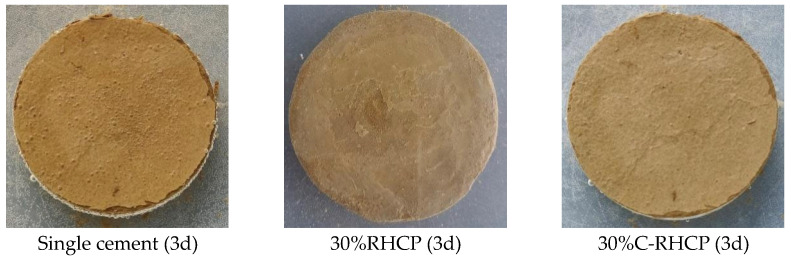

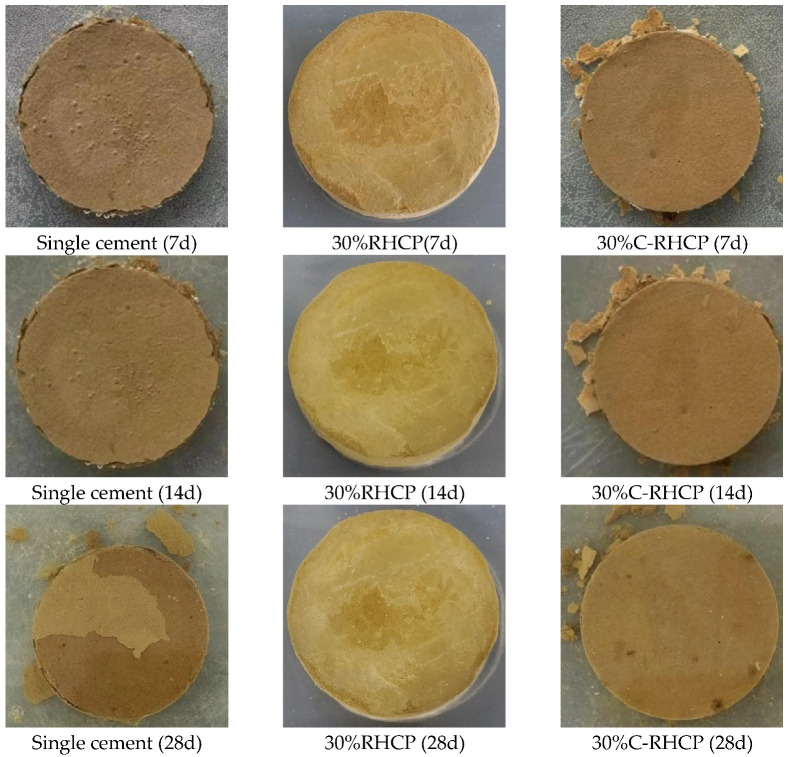
Disintegration process of different specimens at 3d, 7d, 14d, and 28d.

To demonstrate the water stability of solidified sludge, immersion tests were conducted on specimens of sludge solidified with single cement (Group A), 30% RHCP (Group B), and 30% C-RHCP (Group C). The water stability of the three cementitious solidifiers for sludge was evaluated based on the water stability coefficient K and the condition of the samples obtained from the images of their destruction. The water stability coefficient K and the condition of destruction are shown in Table 3 and Figure 13. According to Table 3, the water stability coefficient of Group C for solidified sludge was greater than 1 after 7, 14, and 21 days of immersion. However, Group A’s water stability coefficient was only greater than 1 at a curing age of 7 days, whereas Group B’s coefficient was already below 1 at 7 days. It could be seen that Group C started to deteriorate after 21 days of immersion, whereas Group A began to deteriorate after 7 days of immersion. This indicated that the water stability of C-RHCP-solidified sludge was superior to that of cement and RHCP. The difference in water stability coefficients between Groups A and C was minimal at 28 days of immersion, indicating similar levels of deterioration in both experimental groups. This suggested that C-RHCP-solidified sludge and cement-solidified sludge had comparable water stability after 28 days of immersion.

The disintegration process of different specimens at 3, 7, 14, and 28 days is displayed in Figure 13. For Groups C and A, specimens of SS that had been cured for 7 days, 14 days, and 28 days were found to be intact after 28 days of immersion. Figure 13 visually demonstrates that C-RHCP replacing 30% of the cement significantly improved the water stability of the sludge. For specimens of solidified sludge from Groups A and B, only slight particle detachment was observed on the surface, and no cracks were detected. This was due to the enhanced overall structural integrity and stability of the sludge under the combined action of various solidifiers. The detachment of surface particles may be related to incomplete cohesion during specimen preparation.

#### 3.6.2. Dry and Wet Cycle

**Figure 14:**
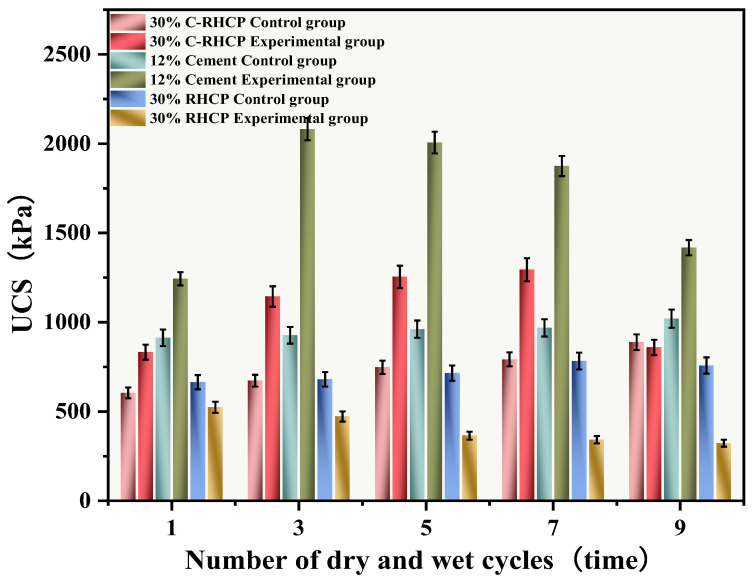
Effect of wet and dry cycles on UCS.

**Figure 15:**
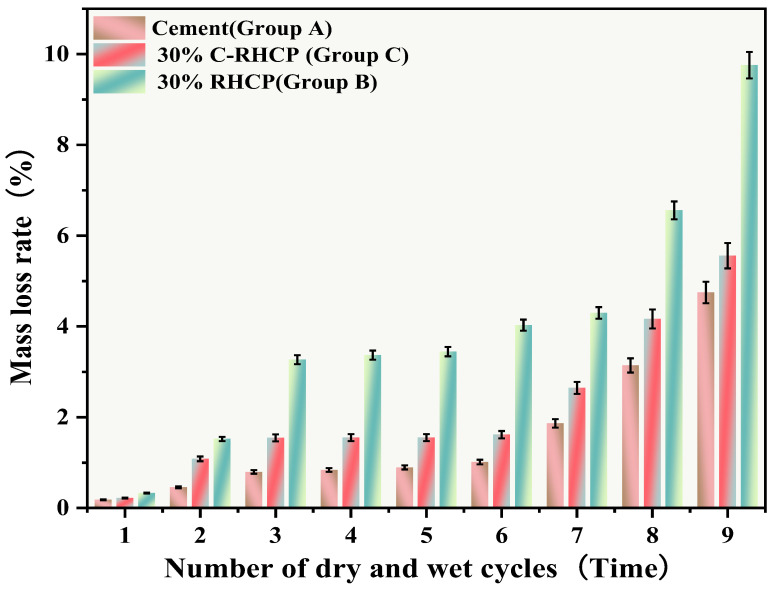
Effect of wet and dry cycling on mass loss rate.

**Figure 16:**
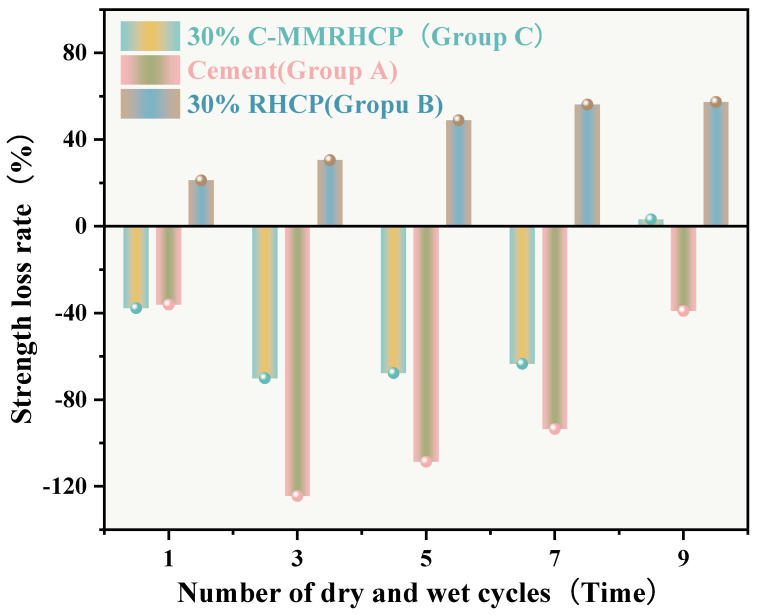
Effect of wet and dry cycling on the rate of strength loss.

The UCSs after 1, 3, 5, 7, and 9 cycles of wet-dry testing are shown in Figure 14. The mass loss rate and UCS loss rate of SS over the cycle number are illustrated in Figure 15 and Figure 16, respectively. The UCS of Group B (30% RHCP) gradually decreased with increasing cycles of wet–dry testing. However, the UCS of Group C (30% C-RHCP) and Group A (single-cement) initially increased with the number of cycles and then decreased. After 1, 3, 5, and 7 cycles of wet–dry testing, Group C exhibited slightly higher strength compared to the control group. When the number of cycles reached 9, its strength relative to the control group showed a slight decrease. This was related to the requirement for sufficient moisture in cement hydration reactions, which were not fully completed during standard curing up to 28 days [61]. This also indicated that as the number of wet–dry cycles increased to a certain extent, there was noticeable strength degradation in the wet–dry testing groups. At this point, the UCS increase due to further reactions in Group C was essentially offset by the negative effects of wet–dry cycling on strength. The cement control group (Group A) absorbed a significant amount of water during the wet–dry cycling process, triggering a series of chemical reactions. This led to the formation of hydration products that bonded the soil particles, thereby enhancing the strength of the solidified sludge. Therefore, after 9 cycles of wet–dry testing, the strength of the experimental group remained higher than that of the control group. After 1, 3, 5, 7, and 9 cycles of wet–dry testing, the UCSs of Group B’s experimental specimens were consistently lower than those of the control group. The presence of clay particles within the soil caused it to undergo drying shrinkage and swelling deformation. When these deformations were excessive, they led to the formation of small cracks at unstable points within the internal structure. These cracks gradually enlarged with an increasing number of wet–dry cycles, ultimately resulting in structural damage to the soil. This was manifested macroscopically as a decrease in strength.

From Figure 15, it can be observed that the mass loss rates of Groups A and C experimental specimens after 1, 3, 5, and 7 cycles of wet–dry testing were all less than 6%. At the same time, there was no apparent detachment observed on the surface of the specimens. This was due to the dense structure and high strength of the solidified sludge, making it difficult for moisture to penetrate into the solidified body, thereby resulting in minimal mass loss. However, for Group B, the mass loss rate after 9 cycles of wet–dry testing approached 10%. This indicated that the non-carbonated RHCP structure was relatively loose and susceptible to erosion by water. In Group C, all specimens remained intact overall, with mass loss rates less than 6%, far below the critical threshold specified by the American ASTM standard (30%). This indicated that using C-RHCP to partially replace cement could significantly improve the durability of solidified sludge. According to Figure 16, the strength loss rate of Group C’s experimental specimens after 1, 3, 5, and 7 cycles of wet–dry testing was negative. At this point, the UCS of the experimental group was slightly higher than that of the control group, which was due to the strength increase caused by the initial wet–dry cycling effect. After undergoing 9 cycles of wet–dry testing, the strength loss rate of the experimental group became positive, and the strength loss increased proportionally with the number of cycles. This indicated that the wet–dry cycling caused a noticeable decrease in UCS for Group C over the cycles. For Group A, after multiple wet–dry cycles, the strength loss rate of the experimental group was negative. Wet–dry cycling was found to contribute to a moderate enhancement in strength. This was because the specimens could fully interact with moisture, allowing reactions to proceed effectively. During the wet–dry cycling and immersion process, the specimens absorbed a considerable amount of water, triggering a series of chemical reactions. Hydration products were produced in these reactions, bonding soil particles and thereby enhancing the UCS of the SS. On the contrary, Group B exhibited positive strength loss rates after multiple wet–dry cycles. It could be found that the specimens exhibited a decrease in strength following the initial wet–dry cycle. Overall, these data suggested that the effectiveness of C-RHCP was superior to both the cement control group and RHCP.

#### 3.6.3. Freeze–Thaw Cycle

**Figure 17:**
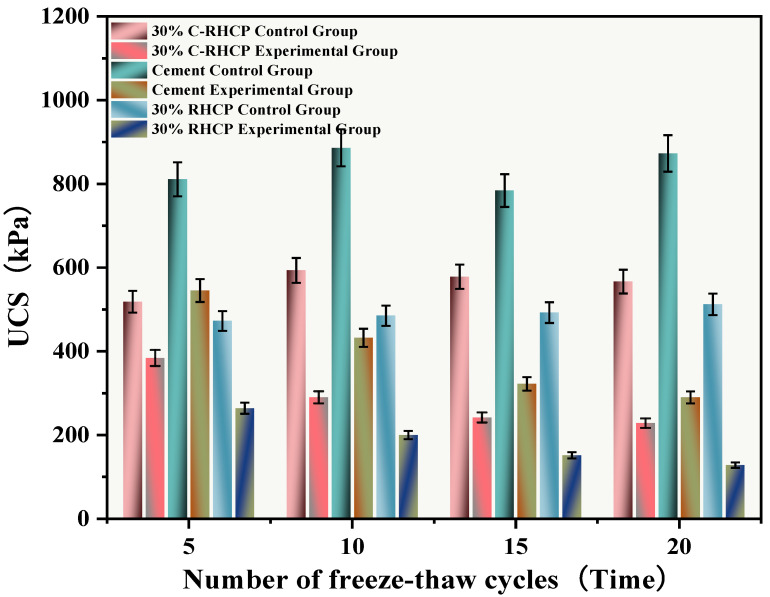
Effect of freeze–thaw cycles on unconfined compressive strength.

**Figure 18:**
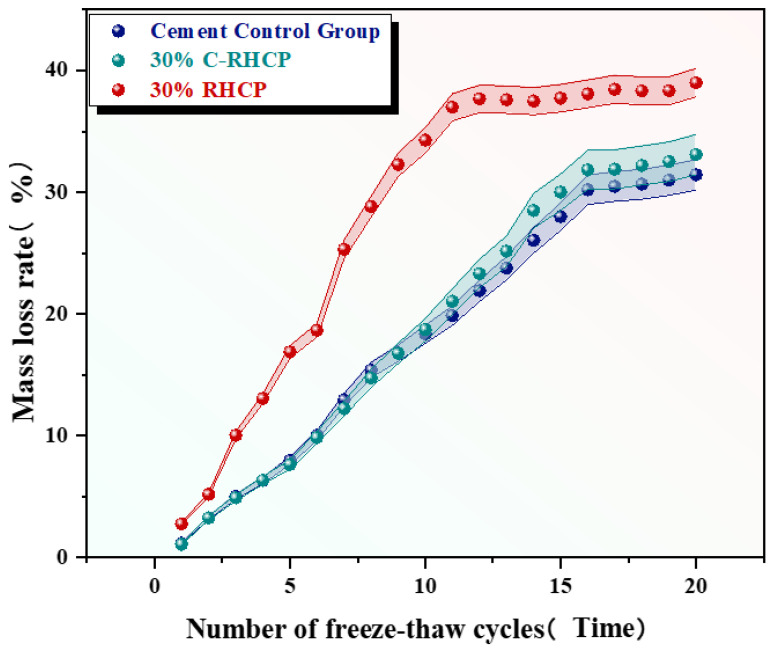
Effect of freeze–thaw cycles on the rate of mass loss.

**Figure 19:**
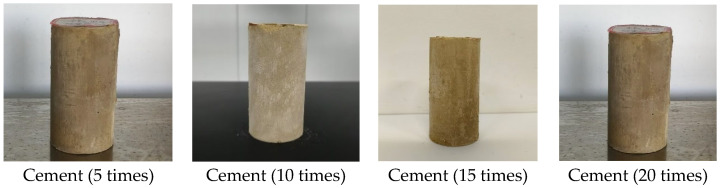

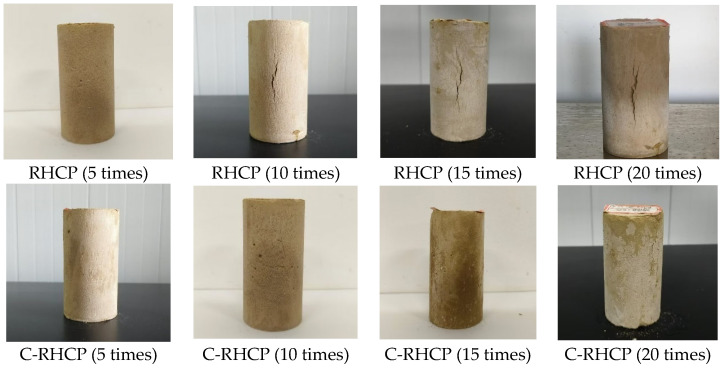
Morphology of samples under different freeze–thaw cycles.

The freeze–thaw cycles simulated the alternating freezing and thawing of moisture on the surface and inside of the solidified sludge, mimicking seasonal temperature changes. After undergoing multiple (5, 10, 15, 20) freeze–thaw cycles, the UCS results of the experimental and control groups were as shown in Figure 17. The mass and strength loss rates of the SS were calculated and are depicted in Figure 18 and Figure 19. From Figure 17, it can be observed that the UCS of the experimental group decreased inversely with the number of freeze–thaw cycles. However, the UCS of the control group increased, and after multiple freeze–thaw cycles, the strength of the experimental group was lower than that of the control group. Compared to the group subjected to 5 freeze–thaw cycles, the strength of the experimental groups subjected to 10, 15, and 20 freeze–thaw cycles decreased by 29.10, 62.81, and 174.41 kPa, respectively. When the temperature dropped below 0 °C, moisture on the surface and inside the solidified sludge condensed, causing expansion [62]. Under significant expansion stress, internal particle bonding in the soil could fail, leading to the formation of micro-cracks, visible as cracks on the surface and internally [63]. When the temperature rose above 0 °C, melting frost allowed moisture to penetrate into the structure through pores or capillary channels on the structural surface. The migration of moisture caused significant changes in structural elements such as pore morphology and particle arrangement in the SS. The alternation of freezing and thawing of moisture within the SS surface and interior weakened its structure, resulting in a decrease in UCS.

From Figure 18, it was observed that the mass loss rates of the cement control group after multiple (5, 10, 15, 20) freeze–thaw cycles were 7.79%, 18.34%, 28.01%, and 31.43%, respectively. The mass loss rate increased progressively with the number of cycles, and the strength loss rate also gradually increased. The mass loss rates of the 30% C-RHCP solidified sludge were 7.62%, 18.70%, 29.99%, and 33.09%, respectively. Compared to the cement control group, the strength loss rates differed only slightly. The mass loss rates of the 30% RHCP solidified sludge were 16.88%, 34.28%, 37.73%, and 38.99%, with strength loss rates higher than those of the cement control group. This was because standard curing conditions were more conducive to the full hydration of cement in the solidified sludge. However, under freeze–thaw cycling conditions, the alternating freezing and thawing of moisture disrupted the bonding and internal structure of the soil. The morphology of the samples under different freeze–thaw cycles is shown in Figure 19. It could be found from Figure 19 that the 30% C-RHCP samples showed loosening and flaking on the surface, accompanied by noticeable layered damage after the cycles. Additionally, the flaking on the surface worsened with an increasing number of cycles. However, the overall appearance remained intact without any obvious cracks. As the number of freeze–thaw cycles increased, significant surface cracks were observed in the 30% RHCP group. This occurred because non-carbonated RHCP contained more pores, making it more susceptible to freeze–thaw damage due to the presence of pore water. In contrast, carbonated RHCP exhibited better resistance to freeze–thaw cycles, as fine calcium carbonate had formed and filled some of the pores, leading to minimal changes in strength.

**Figure 20:**
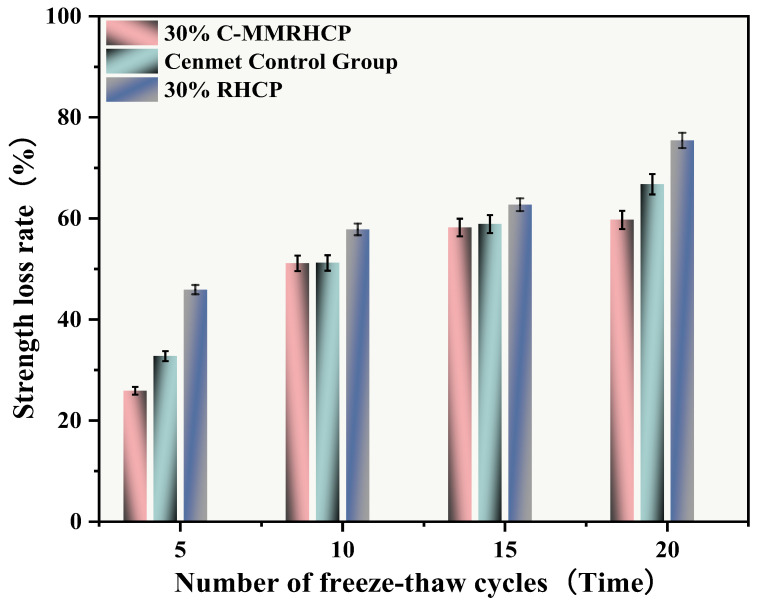
Effect of freeze–thaw cycles on rate of strength loss.

From Figure 20, it was observed that after multiple freeze–thaw cycles (5, 10, 15, 20), the strength loss rates of the cement test group were 32.78%, 51.21%, 58.90%, and 66.77%, respectively. The UCS of the SS decreased gradually with the increase in the number of cycles, and its strength loss rate raised progressively. The strength loss rates of the 30% C-RHCP samples were 25.91%, 51.13%, 58.21%, and 59.72%, respectively. Compared to the cement group, the overall strength loss rate was lower. After 20 cycles, the strength loss rate of the samples with added C-RHCP was 7.05% lower than that of the cement control group. This indicated that it had a certain resistance to the weakening of the UCS of the samples. The strength loss rates of the 30% RHCP samples were 45.91%, 57.83%, 62.71%, and 75.43%. Compared to the cement group’s wet–dry cycles, the freeze–thaw cycling conditions had a particularly significant impact on the strength of the specimens. This destructive effect was manifested macroscopically as a sharp decline in strength, with the test results consistent with those from the wet–dry cycling.

## 4. Conclusions

This study focused on the carbon sequestration performance of RHCP carbonated by microbial methods under different pressures. Additionally, the mechanical and durability properties of sludge consolidated with RHCP and C-RHCP as partial cement replacements were investigated. Based on the experimental results, the following conclusions were drawn:

1. Nonlinear regulation mechanism of pressure on the carbonization process: In the pressurized carbonization system, the solubility of CO_2_ was positively correlated with pressure, but the microbial activity showed an inhibitory effect. The synergistic effect of the two led to a non-monotony response characteristic of carbonization efficiency that first increased and then decreased. The experiment verified that 0.5 MPa was the critical pressure threshold. At this time, the carbonization efficiency reached the peak (59.82 g CO_2_/kg), reflecting the optimal regulation value of pressure parameters in the carbon storage process.
2. The co-evolution law of microstructure and mechanical properties: The microscopic morphology characterization by SEM-EDS confirmed that a dense accumulation layer of nano-micron-scale calcium carbonate crystals was formed on the surface of C-RHCP. Through the pore filling effect and interface strengthening mechanism, the pore structure of the material was significantly optimized, thereby improving its macroscopic mechanical properties. The increase rate of compressive strength compared with uncarbonated RHCP reached 18.7%.
3. The mechanism of bio-chemical synergistic mineralization enhancement: The proliferation effect of interfacial nucleation sites induced by microbial metabolic activity not only accelerated the kinetic process of CO_2_ mineralization into calcium carbonate but also optimized the crystallization orientation and spatial distribution of subsequent hydration products (such as C-S-H gel) by providing heterogeneous nucleation substrates.
4. Long-term performance enhancement via carbonation-activated pozzolanic reaction: Carbonation modification significantly enhanced the reactivity of silicate/aluminate phases in RHCP. These phases interacted with Ca(OH)_2_ to generate amorphous silica gel and secondary C-S-H gel, forming a multi-scale cementitious network that reinforces durability and mechanical strength over extended periods.

The microbial-induced calcium carbonate precipitation (MICP) technology was integrated into the carbonation modification of recycled hardened cement powder (RHCP), and the synergistic effects of microbial activity and pressure on RHCP carbonation efficiency were systematically investigated for the first time. A pressure-microbial-coupled activation system was developed. Compared with the traditional methods, the carbon sequestration capacity and volcanic ash activity of RHCP were both improved. This approach not only provided a low-carbon pathway for RHCP recycling but also established a novel framework for integrating bio-mineralization with industrial waste valorization. Future applications may face challenges in scaling up the microbial pressure system due to variability in bacterial survival under industrial conditions, high operational costs for maintaining controlled pressure environments, and potential long-term durability concerns of carbonated RHCP in harsh geotechnical settings. Additionally, the reliance on pure CO_2_ sources limits its feasibility in regions lacking carbon capture infrastructure. Addressing these constraints will be critical for real-world implementation.

## Figures and Tables

**Table 1:** Main chemical composition of cement and RHCP.

Material	SiO_2_	CaO	Al_2_O_3_	Fe_2_O_3_	K_2_O	MgO	SO_3_
Cement	61.501	17.014	10.388	3.747	2.510	1.293	1.217
RHCP	21.556	63.460	7.396	3.267	0.881	1.782	0.907

**Table 2:** Carbon sequestration amount of C-RHCP under different pressures.

Pressure/MPa	W_1_/%	W_2_/%	(W_1_ − W_2_)/%	Increased Carbon Sequestration/g·kg^−1^
0.1	91.20	83.86	7.34	46.41
0.3	89.98	81.78	8.20	59.16
0.5	90.30	82.03	8.28	59.82
0.7	91.41	83.74	7.67	50.48
0.9	91.91	85.38	6.53	35.37

**Table 3:** Water stability coefficient K.

	Curing Age/Days	7	14	21	28
Group					
Single cement		1.07	0.92	0.96	0.89
30% RHCP		0.96	0.94	0.87	0.82
30% C-RHCP		1.16	1.13	1.10	0.88

## Data Availability

The original contributions presented in the study are included in the article, further inquiries can be directed to the corresponding author.
